# Comparing the Relative Interfacial Affinity of Soft Colloids With Different Crosslinking Densities in Pickering Emulsions

**DOI:** 10.3389/fchem.2018.00148

**Published:** 2018-05-01

**Authors:** Man-hin Kwok, To Ngai

**Affiliations:** ^1^Department of Chemistry, The Chinese University of Hong Kong, Shatin, Hong Kong; ^2^Shenzhen Municipal Key Laboratory of Chemical Synthesis of Medicinal Organic Molecules, Shenzhen Research Institute, The Chinese University of Hong Kong, Shenzhen, China

**Keywords:** Pickering emulsions, microgels, PNIPAM, soft colloids, microgel-stabilized emulsions

## Abstract

Pickering emulsions prepared by various kinds of soft colloids such as the poly(*N*-isopropylacrylamide) (PNIPAM)-based microgels, have been studied for decades in order to fabricate stimuli-responsive emulsions. It has been generally viewed that the interfacial properties of the microgel monolayers and the emulsion stability are dominated by the softness or deformability of the microgel particles. However, there is still no convenient way to characterize the adsorption/desorption energy of the microgels at the interface although this is an essential topic for microgel-stabilized emulsions. This paper presents a novel method for directly comparing the relative interfacial affinity of microgel particles with comparable size but different crosslinking densities, therefore, different softness at the oil/water interface. Typical micron-sized PNIPAM-based microgels were synthesized and used in this study. With advanced fluorescent labeling techniques, we are capable of distinguishing different kinds of microgels in a Pickering emulsion. During vigorous agitation, particles with higher adsorption energy are more likely to be found at the oil/water interface instead of the loosely adsorbed counterparts. By counting the ratio of interfacial area occupied by two microgels, the interfacial affinity of them can be compared. It is found that interfacial affinity of microgels is not only dependent on the softness but also strongly correlated with the core-shell morphology of the microgels, especially the outmost collapsed polymer layer at the interface. This result is consistent with the interfacial morphology model proposed by other researchers. The understanding of the stabilization of such Pickering emulsions can help us to design and develop responsive Pickering emulsions with better controlled stability.

## Introduction

Pickering emulsion was first described by Ramsden (1904) and S. U. Pickering more than 100 years ago (Pickering, 1907). Submicron or micron-sized solid particles like surfactants or amphiphilic polymers, can adsorb at oil-water interfaces, and provided long-term kinetic stability. Such particle-stabilized emulsions are now commonly called Pickering (or Pickering–Ramsden) emulsions. These stabilizing particles are wetted by both phases in the system and they are interfacially active. Unlike the conventional small molecule surfactants, the particle stabilizers are usually considered to be irreversibly adsorbed to the interface and cannot be removed by thermal energy (Schmitt and Ravaine, 2013).

Since the early 1900's studies on solid particles at interfaces, the issue of correlating the properties of individual particles at the interface with emulsion stability has remained largely unexplored until the last couple of decades. Nevertheless, with the advancement in preparation of various kinds of colloidal particles, the topic has attracted so much more attention in physical science research (Binks, 1998; Chen et al., 2007; Li and Stover, 2008; Liu et al., 2008; Tsuji and Kawaguchi, 2008; Richtering, 2012; Destribats et al., 2014; Style et al., 2015). Pickering emulsions retain the basic properties of classical emulsions stabilized by surfactants or proteins so that they can be substituted for classical emulsions in most industrial and technological applications. Moreover, Pickering emulsions offer several remarkable advantages over conventional surfactant-stabilized emulsions, such as high resistance to coalescence and reduced foaming (by hydrophobic particles) (Aveyard et al., 1994). The “surfactant-free” character makes them more attractive in personal care and pharmaceutical applications where surfactants often cause adverse effects such as irritancy and even cell damage (Tang et al., 2015). Therefore, they have received intense attention in the past decade.

Besides hard spherical particles, Pickering emulsions stabilized by soft polymeric particles have also been developed (Ngai et al., 2005, 2006). Particles made of soft matter are able to significantly change their properties when they are triggered by external stimulations, such as temperature (Pelton and Chibante, 1986) pH (Hoare and Pelton, 2004, 2008; Khan, 2007) ionic strength (Saunders and Vincent, 1999) or even magnetic field (Khan, 2008). Therefore, the use of soft particles in stabilizing Pickering emulsions allows a convenient way to prepare responsive emulsions, which are also known as “smart emulsions.” The responsiveness of the soft particles can be transferred to the corresponding Pickering emulsions. The development of such responsive emulsions leads to even more potential applications, for example, biocatalysis (Wiese et al., 2013), oil transportation (Li and Stover, 2008), oil refinery (Brugger et al., 2008), and drug delivery (Frelichowska et al., 2009; Zhang et al., 2010; Chevalier and Bolzinger, 2013).

Whilst soft particles have been demonstrated as being interesting stabilizers for Pickering emulsions, the mechanism and detail of the stabilization given by these soft particles are still not fully understood. In the past few years, many reports studied Pickering emulsions stabilized by soft particles, especially poly(*N*-isopropylacrylamide) (PNIPAM)-based microgel particles (Brugger et al., 2010; Geisel et al., 2012, 2014a,b; Destribats et al., 2013, 2014; Monteillet et al., 2014; Pinaud et al., 2014). The softness or the deformability of microgels has been emphasized to play an important role in the stabilization of emulsions. For example, Destribats et al. (2011) obtained the images of PNIPAM-based microgel particles at the oil/water interface using cryo-scanning electron microscopy (cyro-SEM) techniques. Based on their SEM images, they concluded that microgel particles are often deformed and stretched at the interface. They described the conformation as “fried egg-like structure” and suggested that the deformability of the microgel particles was important in stabilizing the corresponding Pickering emulsions. It is reasonable to attribute the high stability of the emulsion to the deformability of the stabilizers because the flattening of microgel particles would increase the coverage of each particle and form a better and elastic protecting layer.

However, in our recent study, we observed individual, micron-sized microgel particle at the oil/water interface under confocal laser scanning microscopy (CLSM) (Kwok and Ngai, 2016). It was found that the deformation of the overall shape of micron-sized microgel is not significant. Compared with cryo-SEM, CLSM does not offer images with very high resolution but the images can be taken in aqueous solution, the native state instead of high vacuum, dried state of soft particles. Therefore, CLSM is likely a better choice for characterizing these water swollen gel particles. We argued that larger microgel only significantly deform at extremely swollen condition, which refers to the pH-responsive swelling. For PNIPAM microgel without pH-responsiveness, the corresponding deformation might not be significant as shown in cryo-SEM of the sub-micron-sized microgels.

**Figure 1:**
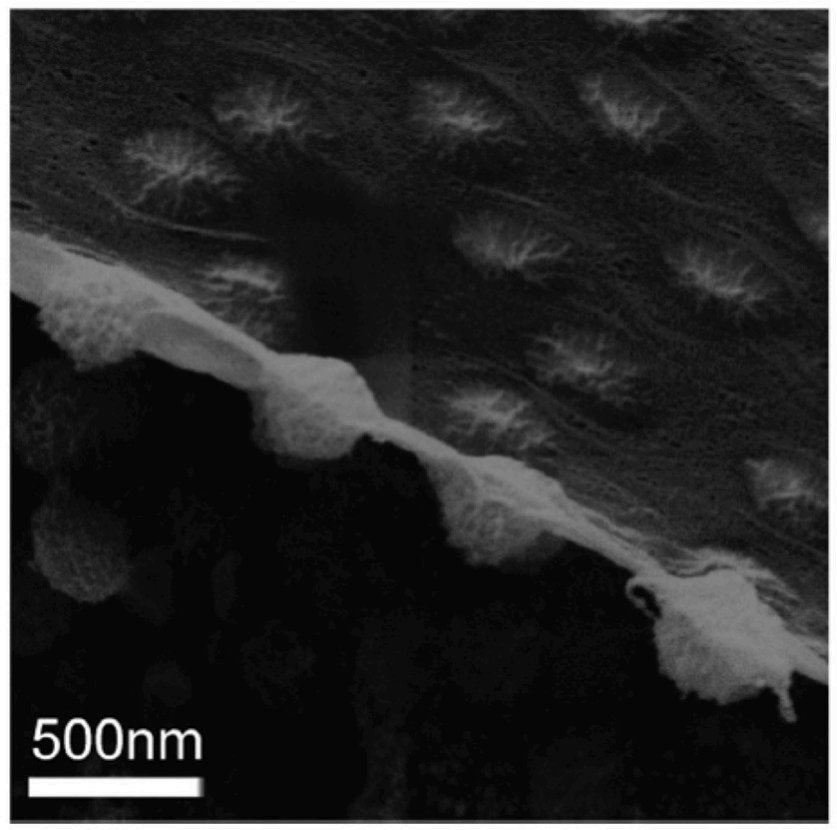
Cryo-SEM image of a fracture water-decane interface populated by PNIPAM microgel particles (Style et al., 2015). Reproduced with permission of The Royal Society of Chemistry.

Besides our CLSM images, Geisel et al. obtained images of microgel-stabilized Pickering emulsions in aqueous state using novel transmission X-ray microscopy (Geisel et al., 2014a). In their images, the main body of the particles do not show any significant flattening or deformation. Nevertheless, deformation near the interface is found. Recently, Style et al. took cryo-SEM images of a fractured water-decane interface populated by PNIPAM microgel particles with good resolution (Style et al., 2015). In this peculiar side-view as shown in Figure 1, soft microgel particles show asymmetric conformations across the interface, with two different sizes and shapes of the particle portions exposed to the two fluids. However, it can be clearly found that the main part of the microgel particle is not significantly deformed. It seems that the oil/water interface in between the particles is covered by a layer of the collapsed polymer which is connected into networks. Their results are consistent with our previous confocal results and the measurement of the elastic modulus of PNIPAM-based microgel particles from other AFM based studies (Hashmi and Dufresne, 2009; Burmistrova et al., 2011; Kwok and Ngai, 2016). Moreover, Zielinska et al. have recently used neutron reflectivity to study the PNIPAM-based nanogels at the water/air interface (Zielinska et al., 2016). They found that the nanogels at the interface have a collapsed polymer layer in contact with air. This collapsed polymer layer has a low water content which is similar to that for a collapsed microgel at temperatures above the volume transition temperature (VTP). However, it is still an open question how the morphology of the microgels and this outermost collapsed polymer layer influence the adsorption/desorption energy of individual microgel particles at the interface which is not easy to be measured. In order to connect interfacial properties between soft particles and emulsion stability, in this study, we have developed a novel method to compare the relative interfacial affinity or surface activity of the microgels with different softness. PNIPAM-based microgels with different crosslinking densities were firstly synthesized and mixed together to stabilize emulsions. By using excess microgel particles, the number of microgels at the oil/water interface was no longer limited by the total number of particles. Instead, the number of a specific microgel sample populated at the interface depended on its affinity to the interface, which directly reflected its desorption energy. With optimized labeling techniques, microgels with different softness within the same emulsion sample can be distinguished clearly. Combining with the deformation model suggested by other literatures, which also matches our previous CLSM images, the stabilization of soft microgels with different softness and morphology on resulting Pickering emulsion can be explained. By keeping the sizes of our microgel samples the same, we found that the interfacial affinities of the microgel stabilizers are not only dependent on the crosslink density but also strongly correlated with the outermost collapsed polymer layer of the microgel in controlling the emulsion stability. The results presented in this paper bring new insights for controlling the stability of Pickering emulsions, particularly using soft colloids as stabilizers.

## Materials and methods

### Materials

*N*-isopropylacrylamide (NIPAM, Fluka) was recrystallized using a 1:1 toluene/n-hexane mixture twice. *N, N*′-Methylenebis-acrylamide (MBA, Fluka) was recrystallized using methanol. Potassium persulfate (KPS, Merck), decane (Sigma Aldrich), methacryloxyethyl thiocarbonyl rhodamine B (RB, Polysciences), and fluorescein sodium salt (FSS, Sigma Aldrich) were used as received. Milli-Q deionized water was used in all the experiments.

### Preparation of PNIPAM microgels

The procedures to prepare micrometer-sized microgel particles with tailored structure and different cross-linker contents have been documented in our previous publication (Kwok et al., 2013). We briefly describe different types of microgels prepared for this work as below.

#### PNIPAM microgels of diameter around 1.4 μm with 10 mg MBA cross-linker

First, 1.0 g of NIPAM, 1 mg of RB, and 0.01 g of MBA were dissolved in 90 ml deionized water and filtered to remove any solid impurities. The solution was then transferred to a 250 ml round-bottomed flask. The solution was purged with nitrogen gas and the solution was stirred in a 43°C water bath for 1 h so that the dissolved oxygen was removed. Then, 0.09 g KPS was dissolved in around 3 ml deionized water and added to the reaction vessel with a syringe for initiation of the polymerization. Once the solution started to turn opalescent, which typically happened within 4–6 min, the temperature was immediately ramped to 60°C with a constant ramp rate of 2°C/min. Finally, the reaction mixture was stirred for 3 h at 60°C. This sample was labeled as L10 (meaning large size of microgel with 10 mg of MBA). Three more microgels with diameters also around 1.4 μm were synthesized with similar procedures but without the RB fluorescent labeling. Their specific conditions and sample names were shown in Table 1. L50A was prepared by the same procedures as L50, except that after the temperature reached to 60°C for 30 min, an extra 10 mg of MBA was added to the reaction mixture. The addition of MBA at the late stage is for cross-linking the dangling chains on the periphery of the microgel particles.

**Table 1:** The experimental conditions for synthesizing L10, L30, L50, L80 PNIPAM micogel particles.

**Sample name**	**MBA content**	**Reaction temperature**	**Volume of solution**
L10	10 mg	43°C	90
L30	30 mg	43°C	110
L50	50 mg	42°C	120
L50A	50 ± 10 mg	42°C	120
L80	80 mg	40°C	140

*Underline values indicates “50 + 10 mg” −50 mg of MBA was used initially and 10 mg of MBA was added in the middle of the reaction*.

#### PNIPAM microgels of diameter around 900 nm with 30 mg MBA cross-linker

Similar to the procedures of synthesizing L30, the monomer solution was prepared, but with a volume of 60 ml and 1 mg of RB dissolved in the solution. After 0.05 g of KPS was added to the reaction vessel at 55°C, the temperature was immediately ramped to 70°C in half an hour. Finally, the reaction mixture was stirred for 3 h at 70°C. This sample was called M30.

All of the synthesized microgels were purified by centrifugation in order to remove any unreacted monomers, oligomer chains and the unreacted initiator. The microgels were purified at a constant maximum centrifugal force of 28,000 g for 1 h. After that, the supernatant was removed and the microgels were dispersed again in deionized water (or microgel solution for concentrating the sample) by stirring overnight. The purification cycle was repeated four times for each of the samples.

### Physical measurements

#### Laser diffraction measurement

Deionized water was used to fill up the sample chamber of the Coulter LS230 laser diffraction size analyser. Background measurements and detector alignment were done by the provided software. Then 1% wt/wt microgel samples were added to the analyzer and the measurements of the particle sizes were performed.

#### Concentration determination

The mass of a clean glass vial was recorded accurately by an analytical balance. After that, about 0.5 mL of the purified microgel sample was transferred to the glass vial, and the total mass of it was measured carefully. Then the glass vial was put in an oven at 150°C to evaporate the water. After the vial was cooled to room temperature, the total mass of the residue and the vial was measured again. Finally, the concentration of the microgel was calculated as a weight percentage.

#### Pickering emulsion stability measurement

0.7 mL of decane was added to 0.7 mL of 1.0% wt/wt microgel solution. Then, the emulsion was prepared by an Ultra Turrax T25 homogenizer (with 10 mm head) operating at 9,500 rpm. After that, the emulsion was placed in a centrifuge for 30 min. The centrifugal force was set at 1,000 g. The centrifugation was repeated until the oil released did not change anymore. Finally, a photo of the emulsion after centrifugation was taken to measure the oil released.

#### Relative interfacial affinity of different microgels

Fifty microliters of decane was added to 1 mL of the 1% wt/wt mixed microgel solution 0. 30 μL of 0.3 mg/mL FSS solution, and 10 μL of 0.075 M sulfuric acid were also added. Then, the emulsion was prepared by the homogenizer operating at 9,500 rpm for 2 min. CLSM images of the emulsion were taken with a Nikon Eclipse Ti inverted microscope (Nikon). The wavelength of the excitation laser for FSS and RB were 488 nm and 543 nm respectively. A 60× (NA = 1.49) oil immersion objective was used. Images were taken from many different portions of the emulsion.

## Results and discussion

### Microgel preparations and characterizations

**Figure 2:**
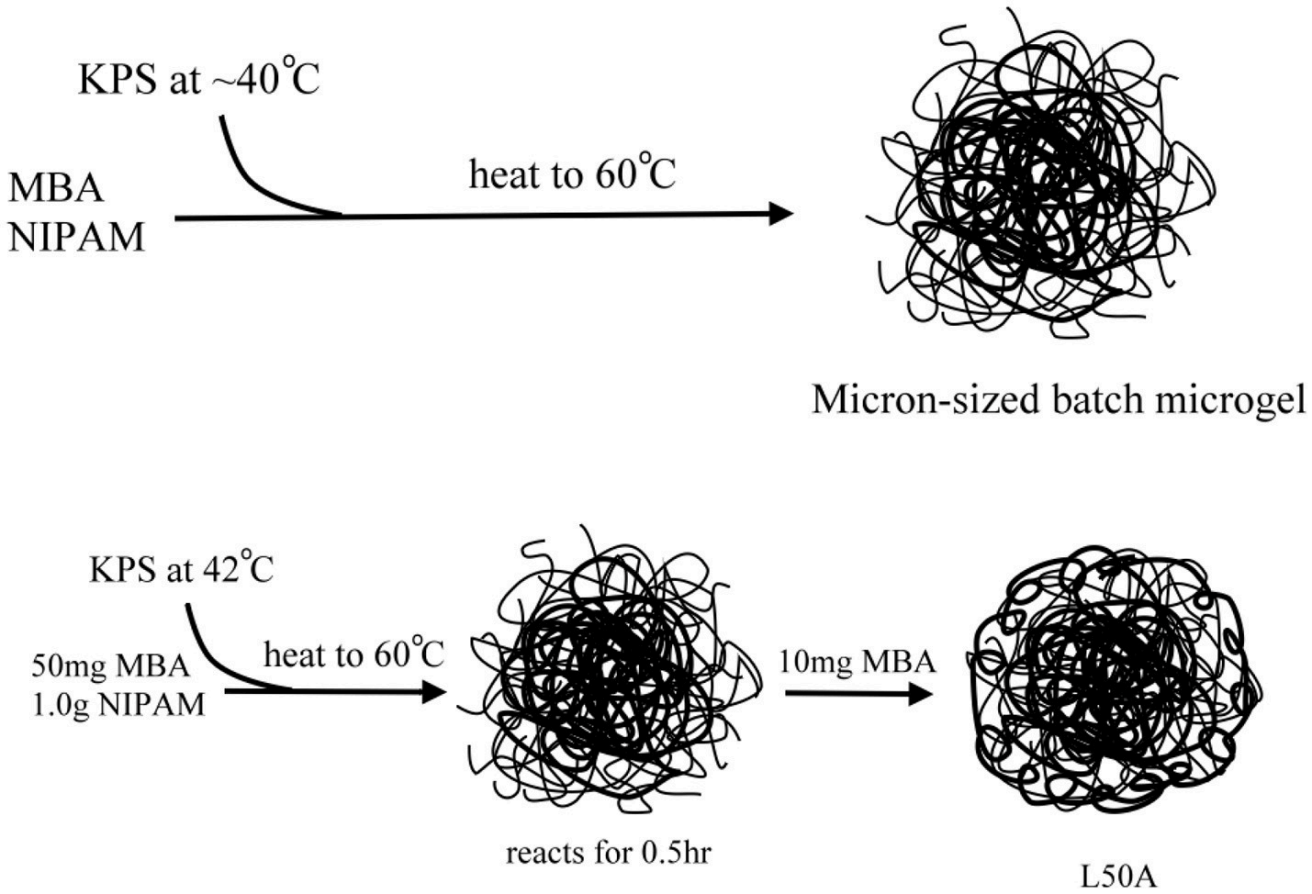
Schematic illustration of the preparation of the micron-sized PNIPAM-based microgel samples.

To synthesize the required microgel particles, surfactant free emulsion polymerization (SFEP), which is also known as precipitation polymerization, was applied. Figure 2 shows schematics of the syntheses. It is found that among numerous synthetic parameters, cross-linker content, nucleation temperature and the total monomer concentration are the key parameters for controlling the particle size. All of the syntheses in this work were based on batch synthesis. The monomers were all added to the reaction mixtures, except for sample L50A. It is worth noting that many reports have indicated that batch polymerization at high temperature can result in a poorly controlled microgel network structure since the cross-linker MBA monomer was commonly incorporated into the microgels faster than the NIPAM monomer. This suggests that the microgel particles prepared in this study would have a core-shell morphology with a highly cross-linked core surrounded a shell of dangling polymer chains. We used only one such kind of particle morphology because the Pickering emulsions stabilized by microgel particles are complicated. Therefore, the comparison of emulsion stabilities was limited to only one variable, the total cross-linker content or softness of the microgels.

For the syntheses of micron-sized microgels with different cross-linking densities (samples named as L10, L30, L50, L80, L50A), temperature-programmed emulsion polymerization was applied. Large PNIPAM particles can be prepared at lower temperature, which is typically around 45°C. However, the yield of the reaction is relatively low and a lot of oligomeric chains, not involved in particle growth, will be formed. Therefore, applying a temperature ramp right after the nucleation state can produce stable micron-sized microgel dispersions with reasonable yield. High cross-linking density on the other hand prevents the microgel from dissolving in water at low temperature. Therefore, it was important to note that the temperature of each synthesis was slightly different. In order to prepare microgels with different cross-linker contents and similar diameter, L10 was prepared at a slightly higher temperature so that the size of it could be reduced and L80 was prepared at a slightly lower temperature. The volume of reaction mixtures was different so that aggregation in the syntheses could be minimized.

**Figure 3:**
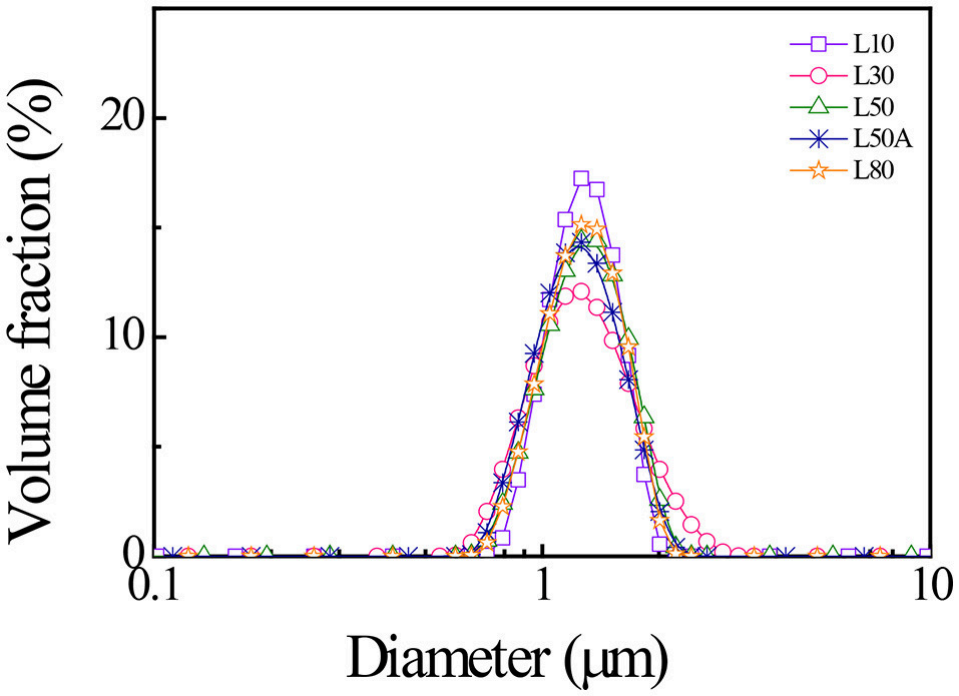
Laser diffraction results of the synthesized micron-sized PNIPAM with different cross-linker contents measured at 25°C.

We used a laser diffraction particle size analyser and dynamic light scattering (DLS) to characterize the diameters of the synthesized PNIPAM microgels. Figure 3 shows the laser diffraction measurements of the microgels. The size distributions of large microgels were very similar, with means around 1.4 μm. In Table 2, the size measurements of all five microgels were summarized. We calculated the thermal responsive swelling ratios (Q) by dividing the diameters measured by DLS D_h_ at 25°C with D_h_ at 40°C. This swelling ratio was affected by the softness of the particles. The swelling ratio increased as the softness of the particle increased. From the diameter swelling ratios shown in Table 2, it was found that the difference in diameter swelling ratios between L10 and L80 were consistent with the corresponding cross-linker content.

**Table 2:** The D_h_, D_LD_ and the corresponding VPT diameter swelling ratios (Q) of the PNIPAM microgels.

**Sample**	**D_h_ (nm) at 25°C**	**D_LD_ (nm) at 25°C**	**D_h_ (nm) at 40°C**	**Q**
M30	960 ± 110	940 ± 270	408 ± 60	2.3
L10	1400 ± 240	1360 ± 260	470 ± 46	3.0
L30	1450 ± 240	1390 ± 420	518 ± 80	2.8
L50	1400 ± 260	1380 ± 320	518 ± 86	2.7
L50A	1350 ± 210	1330 ± 310	504 ± 79	2.7
L80	1370 ± 220	1360 ± 300	522 ± 101	2.6

### Determining the stability of the pickering emulsions stabilized by microgels using centrifugation

For measuring the stability of the microgel stabilized emulsion, centrifugation was applied as it is a widely utilized method to quantitatively measure emulsion stability. The advantage of this method relies in the fact that it is direct and easy to perform. The method determines the maximum pressure which can be withstood by the water thin film between oil droplets before coalescence occurs. This pressure is called the maximum osmotic pressure *P*_*osm*_. Campbell et al. have suggested that this maximum osmotic pressure is a complete analogy with the maximum capillary pressure (Tcholakova et al., 2002). Therefore, emulsions with a higher *P*_*osm*_, are able to resist coalescence for a longer period of time, the emulsions are thus more stable. To calculate this *P*_*osm*_ from the centrifugation data, the following equation was used:

(1) $$\begin{array}{l} P_{osm}=\Delta \rho \;g_{max}\left(H_{oil}-H_{r}\right) \end{array}$$

In this equation, *g*_*max*_ is the maximum centrifugal acceleration; Δρ is the density difference between the oil and water; *H*_*r*_ is the height of the oil released by the centrifugation process, and *H*_*oil*_ is the height of the oil when there is total phase separation.

**Figure 4:**
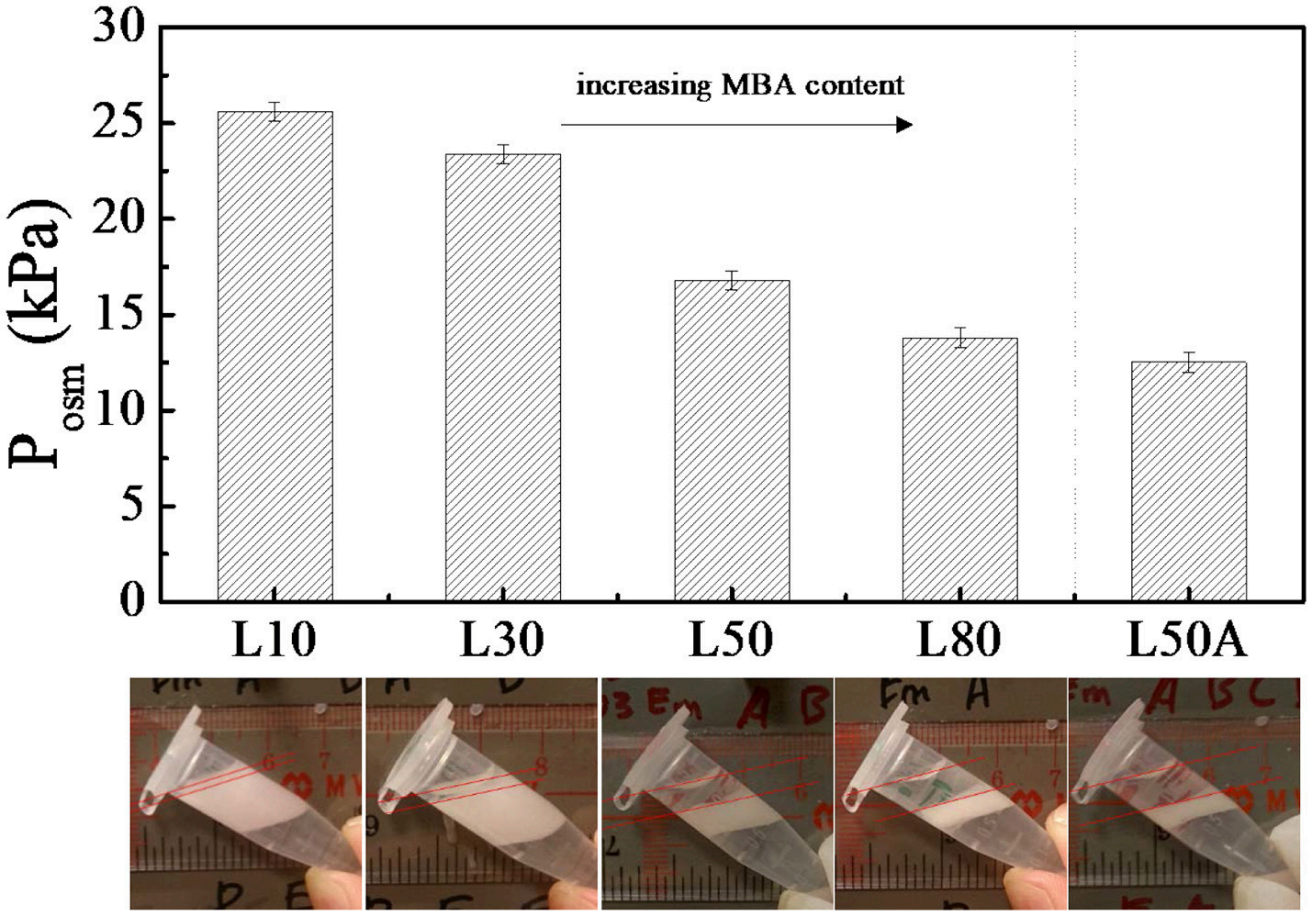
The maximum osmotic pressure (P_osm_) of emulsions stabilized by large microgels with different cross-linker contents. The centrifugation was done at 1,000 g (g is gravitational acceleration). The images below the bars are the corresponding photos of the emulsion samples after centrifugation.

Figure 4 shows the emulsion stability measurements of the large microgel stabilized emulsions after centrifugation at 1,000 g. Although the energy of the particle desorption is usually a few orders larger than the centrifugal potential energy, it was essential to further confirm that the centrifugal force was not large enough to actively remove microgel particles from the oil-water interface. Therefore, the centrifugation was repeated and it was found that the amount of oil released was unchanged after a few centrifugations.

In Figure 4, it can be seen that the stability of the emulsion decreased as the MBA content increased for the microgel stabilized emulsion samples. Note that sample L50A was an exception. By comparing the maximum osmotic pressures of emulsions were stabilized by L50 and L80 with L50A, we found that L50A stabilized emulsions were the least stable. However, L50 and L50A just differed by the surface property and L80 possessed even more MBA content that L50A. We will discuss more about this stability difference in the next section.

### Comparison of surface affinity between microgels with different size

It is often suggested that Pickering emulsion stability is strongly related to the interfacial energy of the particles because they reduce the area of the interface between the two immiscible liquid. Also, the energy is usually a few orders of magnitude larger than thermal energy, which leads to an ultra-strong anchoring of particles at the interface. The energy required when a single rigid spherical particle was desorbed from the interface is given by:

(2) $$\begin{array}{l} \Delta E=\gamma \pi r^{2}{\left(1-|cos\theta |\right)}^{2}\;(ChevalierandBolzinger,\;2013) \end{array}$$

where *r* is the radius of the particle, γ is the surface tension and θ is the contact angle of the particle. This desorption energy is affected by many factors, such as size, contact angle, roughness, etc. If the particles are adsorbed to the interface with higher energy, they form a particle shell around the emulsion droplet with higher strength and the shell is capable of stabilizing the emulsion better. Therefore, the desorption energy is one of the crucial factors in Pickering emulsion stability and this energy can be compared by the surface affinity of the particle. The contact angle of microgel at the oil-water interface was around 40°, which was estimated by the effective contact angle of Richtering's work (Geisel et al., 2012). Therefore, the desorption energy of our larger microgels is around 1.9 × 10^6^ k_B_T.

CLSM is a chosen for this study because the sample preparation is much easier and there is almost no disturbance to the emulsion sample. Also, the emulsion can be visualized in solution instead of vacuum as commonly viewed by electron microscopy. To compare the relative surface affinity of different microgel particles, we mixed two microgel samples, which were labeled differently, and prepared the emulsion with the homogenizer after adding oil. CLSM images were taken and the number of each particle type at the oil-water interface counted. We call this number ratio ϕ. As the diameters of the particles were well-characterized, the relative surface coverage, which is defined to be our relative interfacial affinity Φ can be easily calculated. The method is based on the equilibrium established by the two kinds of microgel and the energy input by the homogenizer. With rigorous agitation, individual microgel particles in the bulk solution are capable of displacing another particle which has been adsorbed at the interface. The probability of this process is depended on their relative desorption energy. A particle with lower desorption energy is less likely to displace a particle with higher desorption energy and vice versa. Therefore, higher interfacial affinity indicated higher desorption energy of the particles.

**Figure 5:**
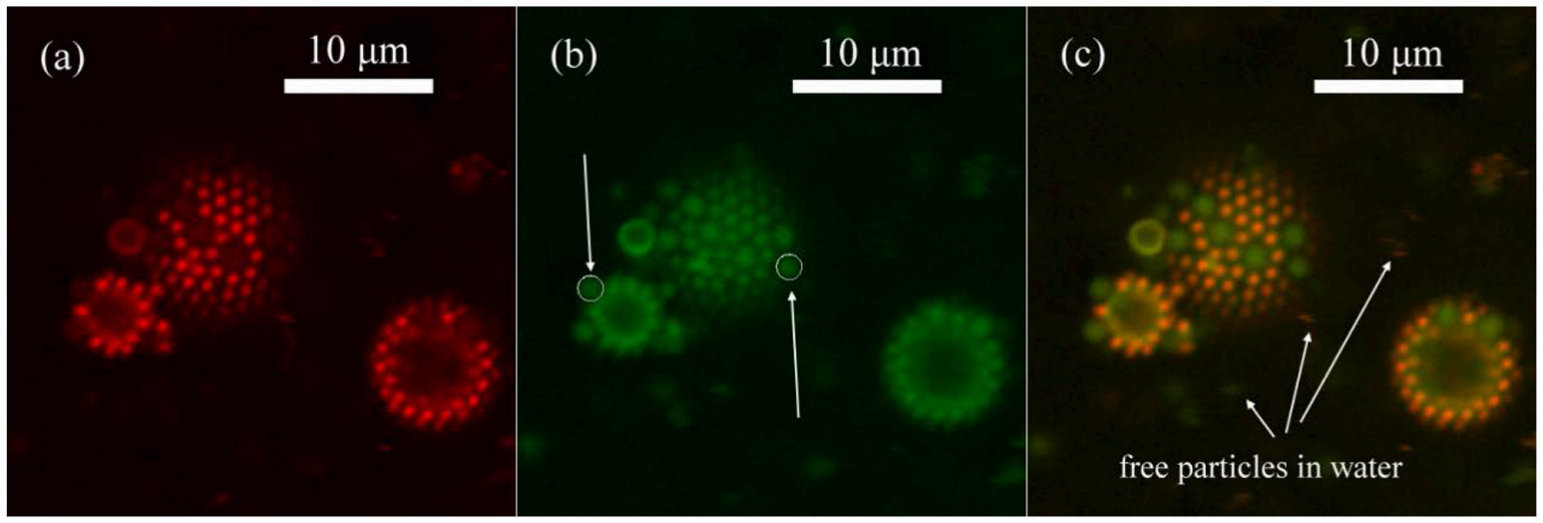
CLSM image of emulsion prepared by M30 and L30 microgels in 1:1 weight concentration ratio in bulk **(a)** Red channel of Rhodamine B fluorescence, which was only given by M30 microgels. **(b)** Green channel of fluorescein fluorescence, which was given by M30 and also L30 microgels. **(c)** Combined image of both channels. Free M30 and L30 microgel particles presented in water, indicating that microgel particles were in excess in the system.

We synthesized microgel M30, which has diameter of around 900 nm and polymerizable red fluorescent dye, RB, was also added in the synthesis. The reason that we used sample M30 instead of L10 in the demonstration was because RB could be much better incorporated into the microgel at high temperature synthesis so that the image quality was better. In Figure 5, we can clearly see that M30 and L30 showed different colors under CLSM. M30 is orange and L30 is mostly green. In the synthesis of M30, RB was added so that the red fluorescent dye was covalently bonded onto the M30 microgel. Next, both of the microgels were labeled by diffusing fluorescein (from fluorescein sodium salt). The adsorption of fluorescein to the microgel was based on the H-bond interaction. (Kwok et al., 2013) Therefore, the two microgels were clearly distinguishable at the oil-water interface. The initial bulk ratio (weight concentration) between M30 and L30 was 1:1. To obtain a statistically valid result, over 4,000 particles were counted from different portions of the emulsion.

In addition, for the smaller emulsion droplet in Figure 5b, the CLSM image was taken near half of the height of the droplet. On the other hand, the CLSM image of the middle sized droplet in Figure 5b was taken near the bottom of the droplet. A white circle of the same size was put onto one L30 particle on each droplet. Obviously, the two L30 particles were identical in either shape or size under CLSM. Therefore, we also could confirm that the main body of the particles were not deformed significantly.

In Figure 5c, we can see that there were free M30 and L30 microgel particles, which were not adsorbed at the interface, in the bulk solution. This is because only a very small amount of oil was used to prepare the emulsion. Both M30 and L30 were in excess while the emulsion was prepared under the vigorous stirring of the homogenizer. In order to further confirm that the current CLSM image results were representing the equilibrium distribution between M30 and L30 on the interface, we prepared the emulsion in four different ways. The initial bulk ratio between M30 and L30 was changed to 2:1 and 1:2, respectively. Also, the emulsion was first prepared by M30 (or L30), then L30 (or M30) was added and the homogenizing process repeated. Table 3 summarizes all five results and the results of (A), (B), (D), and (E) were statistically the same. Result (D) and (E) showed that under homogenization, particle desorption and displacement was allowed. Both M30 and L30 microgel particles were capable of displacing each other from the oil-water interface. Therefore, these particle counting results were not kinetically controlled by the initial adsorption. From result (A) and (B) shown in Table 3, it was confirmed that both M30 and L30 microgels were in excess at an initial ratio of 1:1 and 2:1. It was because the excess particles in the solution did not affect the equilibrium of the adsorption of different particles at the interface. However, from result (C) in Table 3, it was believed that at a M30 to L30 ratio of 1:2, M30 microgel was limited in the system, so that the excess interface was covered by L30 instead. Hence, result (C) in Table 3 was smaller than results (A) and (B).

**Table 3:** Particle counting results of M30 and L30 at the interface.

**Image no**.	**1**	**2**	**3**	**Total**
**(A) M30 TO L30 WT. CONC. RATIO** = **1:1 (# RATIO** = **3.4: 1)**,				
**EMULSION PREPARED BY ONE STEP**				
No. of M30	701	3,776	1,863	6,340
No. of L30	127	672	329	1,128
M30: L30 at interface	5.52	5.62	5.66	5.62 ± 0.05
**(B) M30 TO L30 Wt. CONC. RATIO** = **2:1 (# Ratio** = **6.8:1)**,				
**EMULSION PREPARED BY ONE STEP**				
No. of M30	1,633	1,702	1,380	4,715
No. of L30	290	302	249	841
M30: L30 at interface	5.63	5.64	5.54	5.61 ± 0.05
**(C) M30 TO L30 WT. CONC. RATIO** = **1:2 (# RATIO** = **1.7: 1)**,				
**EMULSION PREPARED BY ONE STEP**				
No. of M30	625	1,459	1,179	3,263
No. of L30	215	513	421	1149
M30: L30 at interface	2.90	2.84	2.80	2.84 ± 0.05
**(D) M30 TO L30 WT. CONC. RATIO** = **1:1 (# RATIO** = **3.4: 1)**,				
**EMULSION PREPARED BY M30 FIRST**				
No. of M30	1,912	1,759	1,089	4,760
No. of L30	351	305	196	852
M30: L30 at interface	5.45	5.77	5.56	5.59 ± 0.17
**(E) M30 TO L30 WT. CONC. RATIO** = **1:1 (# RATIO** = **3.4: 1)**,				
**EMULSION PREPARED BY L30 FIRST**				
No. of M30	1,066	1,455	2,415	4,936
No. of L30	191	261	431	883
M30: L30 at interface	5.58	5.57	5.60	5.59 ± 0.02

*The overall ratio is the average of φ and its weighted standard deviation. The initial bulk # ratio was also calculated based on the assumption that M30 and L30 have the same density*.

We determined the equilibrium number ratio ϕ of M30 to L30 on the oil-water interface to be 5.62 at 9,500 rpm stirring, from result (A) in Table 3. However, it was important to note that small and large particles occupied different areas at the interface. Therefore, to compare their desorption energy obviating the size effect in equation (2), equilibrium interfacial coverage ratio Φ was calculated. This ratio Φ indicated the ratio of interfacial area covered by the two microgels when the exchanging particles established equilibrium with the energy input by the stirring. From the CLSM image in Figure 5, the areas occupied by each M30 particle and L30 particle on the interface were determined to be 0.72 and 1.65 μm^2^, respectively. Therefore, Φ of M30 to L30 was determined to be 2.44, larger than 1. That meant for a given oil-water interface with a certain area, the adsorption of small microgel particles was more energetically favorable than the adsorption of the large microgel particles. Unfortunately, we could not quantify the difference of their desorption energy. It was because we could not quantify the energy which was given by the homogenizer. Nevertheless, this method provided an effective way to qualitatively compare the relative desorption energy of particles per unit area.

### Comparison of interfacial affinity between microgels with different cross-linker contents

**Figure 6:**
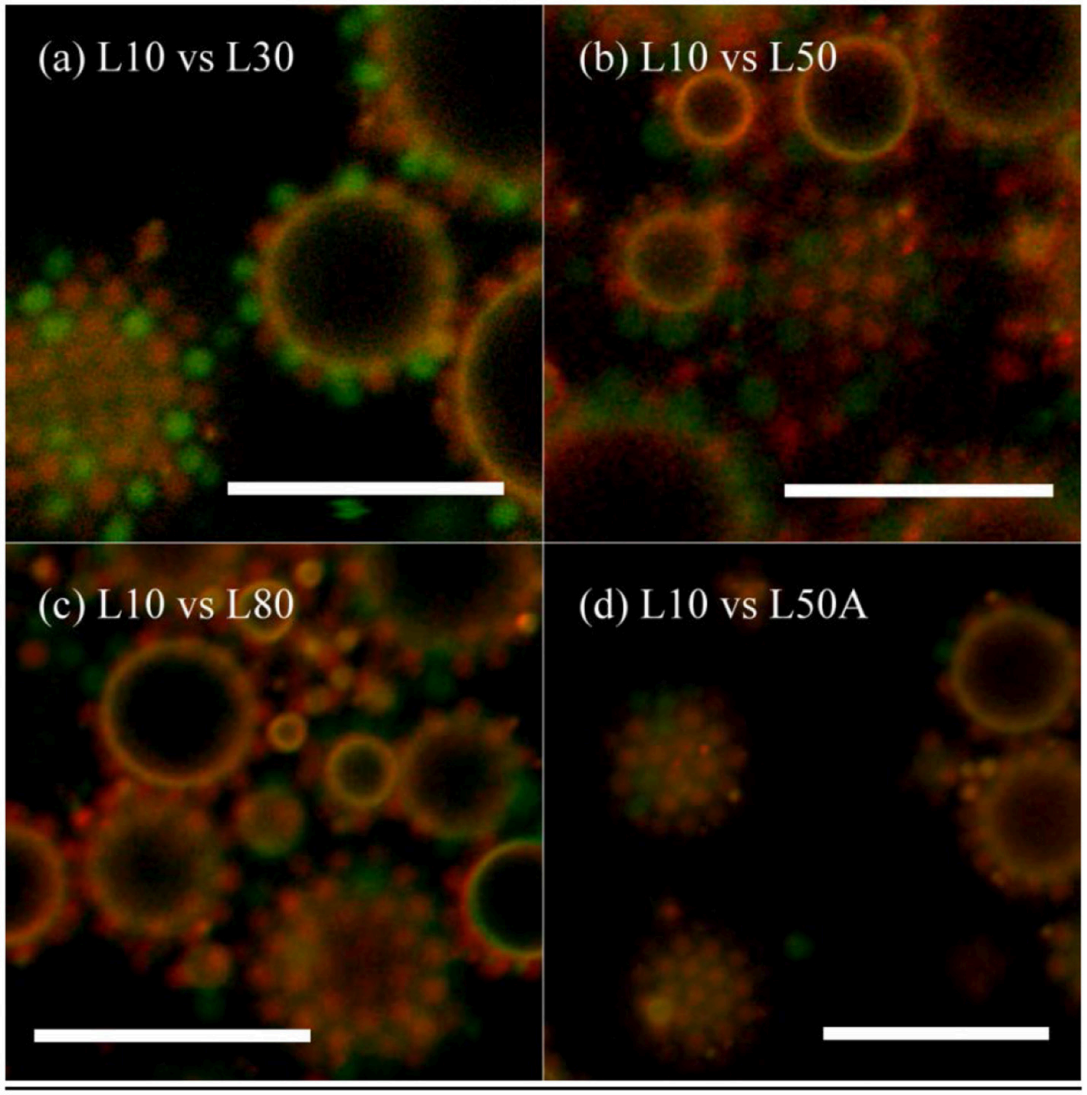
CLSM image of emulsion prepared by L10 and other micron-sized microgels in 1:1 wt. conc. ratio. **(a)** L10 + L30, **(b)** L10 + L50, **(c)** L10 + L80, and **(d)** L10 + L50A. Red Rhodamine B fluorescence was only given by L10. Green fluorescein fluorescence was given both of the particles. The scale bars are all 10 μm.

Similar to the previous comparison of surface affinity between microgels with different sizes, microgels with different cross-linker contents were mixed together and emulsions were prepared by the homogenizer. As we had five micron-sized microgel samples, the interfacial affinity of L10 was compared with L30, L50, L80, and L50A. Although the laser diffraction measurements of these samples were not exactly the same, their diameters in the CLSM images in Figure 6 were similar. Different types of the particles could mix with each other and achieve hexagonal packing on the interface. Table 4 summarizes the particle counting results. As each of these particles occupied almost the same area, we could compare their interfacial affinity simply having the number ratio equaled to the coverage ratio (i.e., φ = Φ).

**Table 4:** Particle counting results of large microgels at the interface.

**(A) L10 MIXED WITH L30**									
**Image no**.	**1**	**2**	**3**	**4**	**5**	**6**	**7**	**8**	**Total**
No. of L10	120	151	192	275	181	130	130	462	1,641
No. of L30	59	76	97	163	92	64	58	243	852
L10: L30 at interface	2.03	1.99	1.98	1.69	1.97	2.03	2.24	1.90	1.93 ± 0.15
**(B) L10 MIXED WITH L50**									
**Image no**.	**1**	**2**	**3**	**4**	**5**	**6**	**7**	**Total**	
No. of L10	318	345	434	243	245	342	295	2,222	
No. of L50	118	131	158	92	102	126	112	839	
L10: L50 at interface	2.69	2.63	2.75	2.64	2.40	2.71	2.63	2.65 ± 0.11	
**(C) L10 MIXED WITH L80**									
**Image no**.	**1**	**2**	**3**	**4**	**Total**				
No. of L10	708	897	837	846	3,288				
No. of L80	190	248	244	240	922				
L10: L80 at interface	3.73	3.62	3.43	3.53	3.57 ± 0.12				
**(D) L10 MIXED WITH L50A**									
**Image no.**	**1**	**2**	**3**	**4**	**Total**				
No. of L10	855	747	745	723	3,070				
No. of L50A	232	193	204	198	827				
L10: L50A at interface	3.69	3.87	3.65	3.65	3.71 ± 0.10				

*The overall ratio is the weighted average of Φ and its weighted standard deviation*.

**Figure 7:**
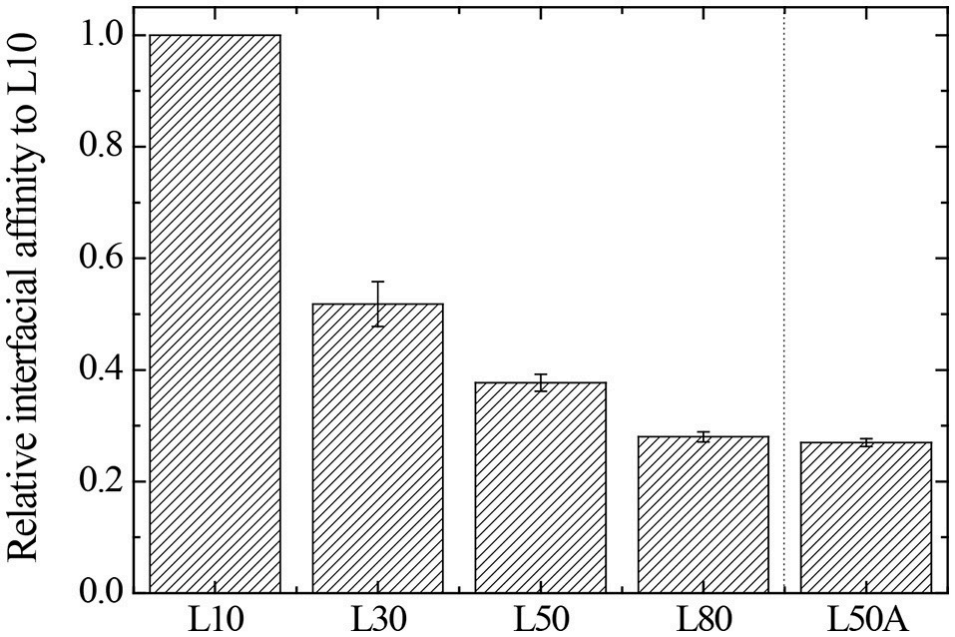
The relative interfacial affinity of micron-sized microgels compared to L10.

According to the results in Table 4, when the cross-linker content of the microgel increased from 30 mg (L30) to 80 mg (L80), their interfacial affinity relative to microgel with 10 mg cross-linker (L10) decreased. More importantly, when we looked at the interfacial affinity of L50A relative to L10, it was smaller than that of L50 and L80. The calculated relative interfacial affinity is shown in Figure 7. The relative interfacial affinity is defined to be the reciprocal of the coverage ratio Φ. Note that the relative interfacial affinity of L10 was by definition set to be 1.

### Stability of microgel stabilized pickering emulsions: effect of cross-linker content

**Figure 8:**
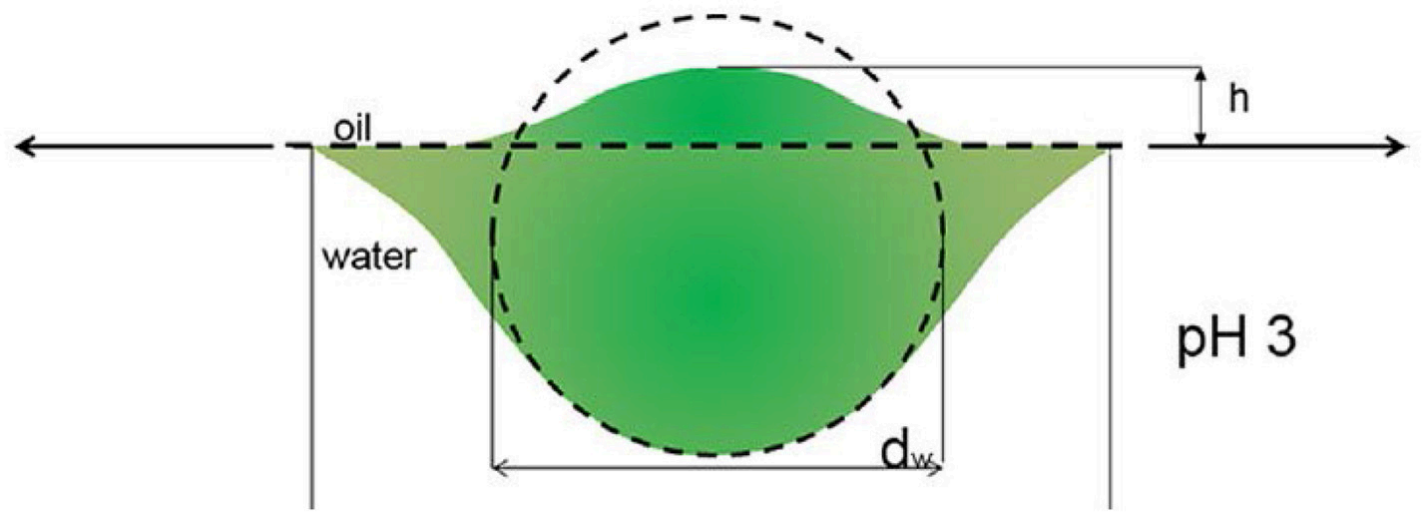
The conformation of microgel particle at oil/water interface proposed by Geisel et al. (2012) Reprinted with permission from. Copyright (2012) American Chemical Society.

Here, we focus the discussion on the effect of cross-linker content. The stabilities of the emulsions showed the same dependence on cross-linker content of microgel (L10, L30, L50 and L80). The stability of the resulting emulsion decreased as the cross-linking content of the stabilizing microgel increases. The main body of our micron-sized microgel particles (cross-linker content between 1.0 and 7.4% wt/wt) were not significantly deforming at the interface. Therefore, we apply the conformation proposed by Geisel et al. in our discussion and focus at the periphery, the collapsed polymer layer at the interface (Geisel et al., 2012). Figure 8 shows a schematic illustration of the proposed conformation of microgel particle and the outermost collapsed polymer layer at the interface.

As mentioned above, in microgel batch synthesis, the cross-linker MBA was more reactive than the NIPAM monomer. As a result, the cross-linking density of the inner part of the microgel is higher. Also, the cross-linking density decreases gradually to the periphery of the particle. Here, we suggest that microgels with less cross-linker content also have a more deformable periphery. Therefore, they deformed more at the oil/water interface. Then, each of them was capable of covering and replacing more area at the interface. These outermost collapsed polymer chains on one hand act as anchors and help the particle attachment to the interface. On the other hand, because of low water content and the strong inter—and interapolymer interaction, the collapsed polymer layers at the interface also have a higher elastic modulus compared to the swollen microgels. As a resulting, the desorption energy of these particles, which has softer outer collapsed polymer layer, is higher.

In the previous section, the relative interfacial affinity was compared to the desorption energy of each of our microgel samples. Therefore, the desorption energy decreased from L10 to L80 gradually. It is consistent with our hypothesis and the stability measurement.

**Figure 9:**
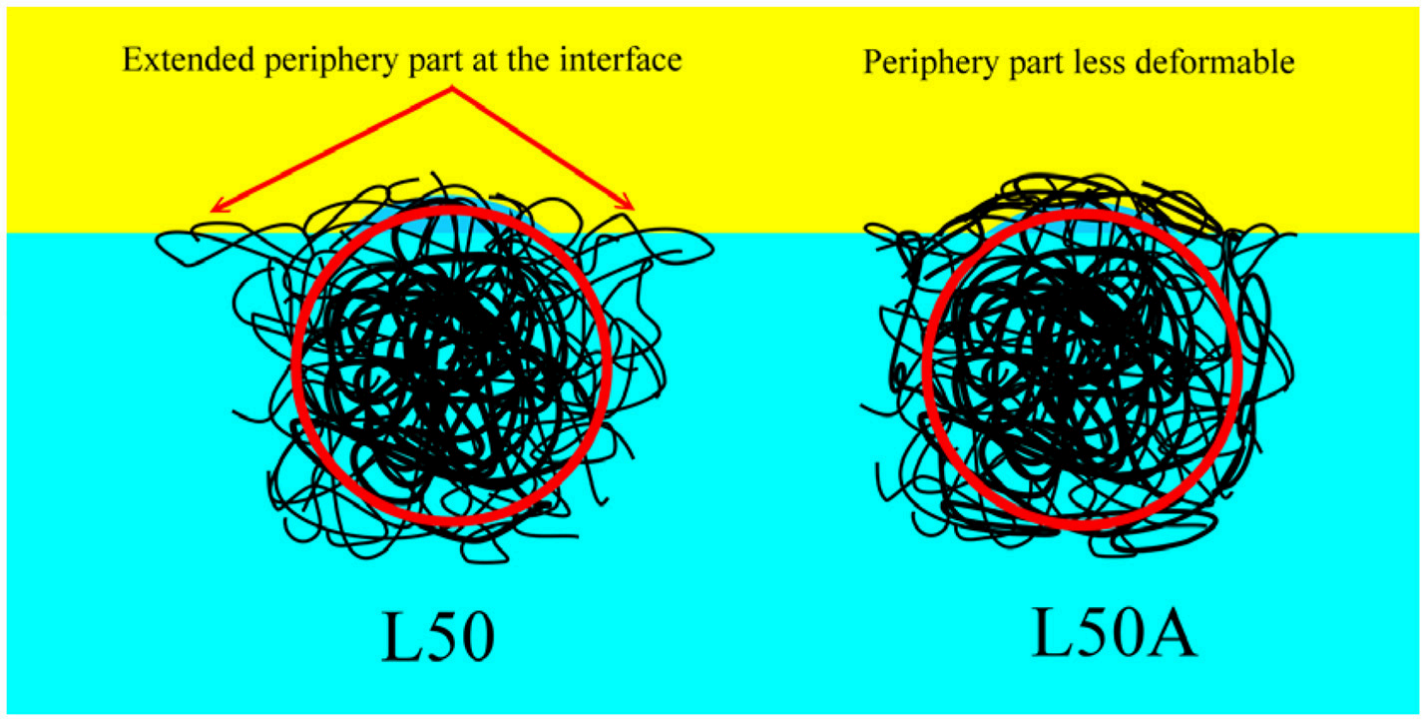
The schematic illustrations of microgel L50 and L50A at oil/water interface. The red circles indicate the part which was labeled effectively by fluorescent dye. The soft periphery part and the main body of the microgel were not drawn in scale.

However, in order to verify this hypothesis, we prepared L50A. As mentioned above, the extra 10 mg of cross-linker MBA was not involved in the particle growth at such low monomer concentrations. Therefore, it changed the microgel particle surface property by cross-linking some of the surface dangling chains. It decreased the deformability of the particle surface and outer portion collapsed polymer layer. It is very important to note that the overall deformability indicated by the thermo-responsive swelling ratios of L50 and L50A were very similar as they were synthesized by the same procedures. From the relative interfacial affinity results, the 10 mg of extra cross-linker significantly reduced the interfacial affinity of the L50A microgel. It is important to point out that the overall cross-linker content of L80 was 33% higher than that of L50A. These interfacial affinity results are also consistent with the stability measurements of the emulsions. In Figure 9, the schematic illustrations of microgel L50 and L50A at oil/water interface are shown. We show that if the outermost collapsed polymer layer portion of the particle was cross-linked, the desorption energy is lower compared to its counterpart, verifying our hypothesis.

## Conclusions

We have demonstrated a new approach for comparing the relative interfacial affinity of soft colloids at the oil/water interface. Microgel samples demonstrated the good confocal image quality. By changing the preparation procedures and the amount of the microgels, it has been confirmed that the final ratio of interfacial particles is not kinetically controlled by the initial adsorption. Once the amount of oil is limited, the ratio is not affected by the amount of particles. Therefore, the interfacial coverage ratio of different microgels derived by this number ratio is capable of representing the relative affinity of the particles. The method was applied to study the effects of cross-linker content and surface deformability on the corresponding microgel-stabilized Pickering emulsions. It was found that microgels with less cross-linker content have higher interfacial affinity and better emulsion stability. Furthermore, the effect is more pronounced for the outermost collapsed polymer layer of the microgel. This result is consistent with the interfacial morphology proposed by other researches and provides direct connection between the deformability and the corresponding Pickering emulsion stability. The improvement in understanding the mechanism of soft colloids stabilized Pickering emulsions will be beneficial for further development of responsive Pickering emulsions with well-controlled stability and performance.

## Author contributions

TN: conceived and managed the research. MK: performed the soft colloids synthesis, emulsion fabrication, and characterizations. TN and MK: reviewed the results and provided the technical guidelines. TN and MK: wrote and drafted the article. TN and MK: reviewed and approved the article.

### Conflict of interest statement

The authors declare that the research was conducted in the absence of any commercial or financial relationships that could be construed as a potential conflict of interest.
